# A damped precipitation‐driven, bottom‐up model for deer mouse population abundance in the northwestern United States

**DOI:** 10.1002/ece3.3598

**Published:** 2017-11-15

**Authors:** Irene L. Gorosito, Richard J. Douglass

**Affiliations:** ^1^ Departamento de Ecología Genética y Evolución Facultad de Ciencias Exactas y Naturales Universidad de Buenos Aires Buenos Aires Argentina; ^2^ Instituto de Ecología Genética y Evolución de Buenos Aires Consejo Nacional de Investigaciones Científicas y Técnicas Buenos Aires Argentina; ^3^ Montana Tech of the University of Montana Butte MT USA

**Keywords:** bottom‐up regulation, deer mouse, mesic habitats, plant productivity, social limit

## Abstract

Small‐mammal population densities can be regulated by bottom‐up (food availability) and top‐down (predation) forces. In 1993, an El Niño Southern Oscillation event was followed by a cluster of human hantavirus with pulmonary syndrome in the southwestern United States. An upward trophic cascade hypothesis was proposed as an explanation for the outbreak: Increased plant productivity as a consequence of El Niño precipitations led to an unusual increase in distribution and abundance of deer mice (*Peromyscus maniculatus*; reservoir host of Sin Nombre virus). Could such drastic events occur in mesic habitats, where plant productivity in response to climate conditions is likely to be much less dramatic? In this work, we investigate to what extent deer mouse populations follow a precipitation‐driven, bottom‐up model in central and western Montana and discuss important conditions for such a model to be possible. We found positive correlations between deer mouse abundance and on‐the‐ground measured plant productivity with a several‐month lag in three of six study sites. This effect was weaker when deer mouse populations were more abundant, indicating density‐dependent effects. Dispersal resulting from territoriality may be important in attenuating local density increments in spite of high food availability. In addition, there is evidence that population abundance in the study area could respond to other abiotic factors. In particular, precipitation in the form of snow may reduce deer mice survival, thus compensating the benefits of improved plant productivity. Deer mouse populations in Montana study sites follow complex dynamics determined by multiple limiting factors, leading to a *damped* precipitation‐driven bottom‐up regulation. This prevents dramatic changes in rodent abundances after sudden increments of food availability, such as those observed in other regions.

## 1 INTRODUCTION

How small‐mammal population densities are controlled has been widely argued for a long time. Most works have focused on the effects of food availability and predation, which are referred as bottom‐up and top‐down forces, respectively. Support for both is found early in the literature. For example, Lack (1954) showed that vegetable food was the major factor controlling various rodent populations' density which in turn led to predator–prey cycles. On the other hand, Pearson (1966, 1971) observed *Microtus californicus* populations were limited by carnivorous predation, which determined the amplitude and synchronization of abundance cycles. More recently, Prevedello, Dickman, Vieira, and Vieira (2013) conducted a meta‐analysis of food supplementation studies and concluded that both bottom‐up and top‐down forces are important for the regulation of populations. However, intrinsic factors such as intraspecific competition could also be relevant for controlling population densities (Conley, 1976). Socially intolerant individuals tend to disperse when density increases, keeping populations locally stable (Krebs, Gaines, Keller, Myers, & Tamarin, 1973; Myers & Krebs, 1971). In situations of extreme densities, even survival and reproduction can be diminished by physiologically driven behavioral changes (Christian, 1950, 1978; David, 1978). Altogether, the complexity of population regulation makes hard to predict how climatic events and other abiotic factors can impact on rodent abundance and dispersal.

Human health risks associated with disease‐bearing species are likely to be triggered by sudden changes in rodent populations. In 1993, a cluster of Hantavirus pulmonary syndrome (HPS) cases caused by Sin Nombre virus (SNV) occurred in the southwestern United States. It was hypothesized that precipitation associated with an El Niño Southern Oscillation event produced increased plant productivity and, consequently, deer mouse (*Peromyscus maniculatus*, the reservoir host of SNV) abundance increased after a several‐month lag in locations where they previously were absent or rare and where plant productivity had previously been extremely low (Dearing & Dizney, 2010; Glass et al., 2002). The concept that the increased abundance and wider distribution of deer mice in the southwestern United States occurred in response to increased plant productivity was called *the trophic cascade hypothesis* (Parmenter, Brunt, Moore, & Ernest, 1993; Yates, Mills, Parmenter, & Ksiazek, 2002). This increase in the deer mouse population may have increased rodent‐to‐rodent transmission of SNV that ultimately spilled over to humans (Mills, Ksiazek, Peters, & Childs, 1999).

This bottom‐up precipitation‐driven process used to explain HPS cases is still discussed in relation to variability among habitats and climatic regimens (Glass 2000; Mills, 2005; Loehman et al., 2012). Deer mice are absent or not abundant in many areas of the normally arid US southwest during typical dry years. In this region, dramatic changes in plant productivity after an El Niño event may produce habitats more appropriate for deer mouse populations, at least temporarily. Studies in the southwestern United States demonstrated the relationship between vegetation growth and deer mouse abundance 1 year later (Engelthaler, 1999; Glass et al. 2000; Glass et al., 2002, 2006; Glass, Shields, Cai, Yates, & Parmenter, 2007). In addition, evidence of a delayed relationship between precipitation and SNV prevalence in deer mouse populations, likely associated with increased plant productivity, has been found in the Channel Islands in California (Orrock, Allan, & Drost, 2011).

In contrast, in the mesic parts of the US west, deer mouse populations tend to be nearly ubiquitous, although at varying abundance regardless of climatic changes (Douglass & Vadell, 2016; Douglass et al., 2001). Mesic habitats (coniferous forests, grasslands, and sagebrush) present situations where plant productivity in response to climate conditions is likely to be much less dramatic than in the arid US southwest. Consequently, the response of deer mouse populations to changing climatic conditions in mesic areas is likely to be less pronounced than those seen in the arid southwest. In addition, predator richness has also been identified as a factor regulating rodent populations independently of precipitation, even in habitats where productivity is strongly affected by precipitation (Orrock et al., 2011). Therefore, the bottom‐up model cannot be generalized straightforwardly to other contexts.

Because the emergence of SNV is linked to changes in climate (Carver et al., 2015), it is important to clarify the link between deer mouse population abundance and plant productivity in the northwestern US where deer mouse populations are persistent. In a study on the effects of climate on deer mouse populations, Luis, Douglass, Mills, and Bjørnstad (2010) showed that deer mouse population dynamics at one location in western Montana were correlated with precipitation, time of precipitation, and temperature after 0‐ to 5‐month lags. However, similar correlations were not found at another location in western Montana (Luis pers. com.). Moreover, Loehman et al. (2012) found that remotely sensed plant productivity provided limited predictive information regarding deer mouse abundance on two sampling grids on which Luis et al. (2010) found climate effects in Montana. These contradictory observations could indicate that remotely sensed plant productivity may not accurately account for available biomass at the scale of single 100 × 100‐m sampling grids.

Our primary objective was to assess whether deer mouse populations follow a precipitation‐driven bottom‐up model in central and western Montana. In so doing, we identify important assumptions of the hypothesis and provide potential explanations for variable results among sampling sites. For this purpose, we evaluated the relationship between deer mouse abundance and various environmental characteristics. In particular, we focus on the response to on‐the‐ground measured plant productivity after various time lags, and we investigate density‐dependent effects.

## 2 MATERIALS AND METHODS

### 2.1 Study area and sampling design

We used deer mouse trapping data based on 850,000 trap nights and environmental data collected at six sites in central and western Montana between 1994 and 2010. Sampled sites included Cutbank, Polson, Cascade, Gold Creek, Wisdom, and C.M. Russell Wildlife Refuge (Douglass, Van Horn, Coffin, & Zanto, 1996). Locations ranged in elevation from 738 to 2,146 m and comprised four habitat types: grassland, sagebrush, meadow, and subalpine fir. For a detailed habitat description, see Douglass et al. (2001). We sampled three grids per site over 12–17 years (Table 1). Except for high‐altitude grids (>1,590 m), deer mice were present during all sampling periods at all locations (Douglass & Vadell, 2016).

**Table 1:** Geographic characteristics and sampling periods for the six study sites in Montana

Location	Range of elevation (in meters)	General habitat	Sampling years	Sampling months
Cascade	1,396–1,415	Grassland	1994–2010	January–December
Cutbank	1,216–2,146	Grassland	1994–2005	May–October
CM Russell	738–927	Forest	1994–2005	January, May–October
Gold Creek	1,591–1,598	Forest, meadow	1994–2005	May–October
Polson	811–915	Sagebrush	1994–2010	March–November
Wisdom	1,957–2,146	Forest	1994–2005	May–October

bg, bare ground; dl, duff litter; fo, forbs; gra, grass; lich, lichens; ro, rock; shr, shrubs.

Trapping and animal handling followed Douglass et al. (1996), according to Mills et al. (1999), and approved by the University of Montana Animal Use Committee, approval #011‐04RDTECH‐021304. We livetrapped for three nights in each sampling period. All animals were marked with ear tags, and sex, breeding condition, weight, and presence of scars were recorded. Blood samples were collected from deer mice at two of the three grids at each site, with the third grid acting as a control grid to determine the effect of blood collection on deer mice (Douglass, Kuenzi, Wilson, & Van Horne, 2000). We released all animals back to the grid on which they were captured.

### 2.2 Vegetation sampling

Each September, after seed ripening, we measured plant cover at 30 randomly selected plots on each grid. We used a one‐half‐meter point frame with 10 rods and recorded the contacts with bare ground, rock, mosses, lichens, duff litter, grasses, forbs, and shrubs. We clipped and placed all herbaceous matter in individual paper bags from each 0.1 m^2^ plot for drying. We also recorded the maximum height of shrubs contacting or overhanging the frame. Herbaceous matter was dried and weighed to determine productivity, beginning in 2002.

### 2.3 Statistical methods

The vegetation variables were determined for each grid as the average number of contacts for bare ground (bg), rock (ro), moss (mo), lichens (lich), duff litter (dl) , grass (gra), forbs (fo), and shrubs (shr) and the average of maximum shrub heights (avshr). Biomass (biom) was calculated as the dry weight per sampled area unit. All the statistical methods described below were performed using the software R (R Core Team, 2016).

Correlation among variables may be underestimated if their distributions are too different in shape (Goodwin & Leach 2006). Therefore, vegetation variables were either logarithmically or square‐root transformed to obtain more symmetrical distributions. Pearson correlation coefficients among transformed variables were <0.33, except between shr and avshr, for which it was 0.51. Collinearity between variables is undesired as it can lead to larger standard errors in parameter estimates. Therefore, the variable avshr was fitted on a linear model in terms of shr, and the residuals were used instead of the original values (i.e., the uncorrelated part of the variable). The remaining variables were centered by subtracting their corresponding mean values after the transformation.

We were interested mainly in the effect of productivity, measured by biomass, on deer mouse abundance, estimated as minimum number alive (MNA). MNA estimates of population size at each sampling period were calculated as the sum of all animals captured during that period, plus the number of individuals that were captured during at least one previous and one subsequent sampling period, but not during the current period (Chitty & Phipps, 1966). Because biomass was not measured from 1994 to 2001, we fitted a linear regression model to extrapolate biomass from point‐frame cover values. We used all habitat cover measures except biom as explanatory variables. We ranked the full model and all its nested models based on the Akaike information criterion corrected for finite sample size (AICc; Burnham & Anderson, 2002). The best model (lowest AICc) included the variables: bg, fo, and gra (Table 2) and was significantly better than any other model (ΔAICc ≥ 2). Consequently, the model using cover of bg, fo, and gra was used to extrapolate biomass. To determine the error in biomass extrapolation, we made a leave‐one‐out cross validation. This procedure simulates the extrapolation on known data, providing an estimate of the expected extrapolation error (Burnham, 1983). We also considered the uncertainty in regression coefficients. Therefore, extrapolated biomass errors were calculated as (σ^2^ + δ^2^)^1/2^, where σ is the regression error and δ is the cross‐validation error.

**Table 2:** The results of models tested to determine best overall for extrapolating biomass based on their Akaike information criterion corrected for small sample size (AICc)

Candidate model	AICc	ΔAICc	Weight
**bg + fo + gra**	**32.1**	**0.00**	**0.293**
bg + fo + gra + dl	34.5	2.48	0.085
bg + fo + gra + lich	34.9	2.81	0.072
bg + fo + gra + shr	35.0	2.91	0.068
bg + fo + gra + ro	35.1	3.00	0.065

The most parsimonious model shown in bold font (ΔAICc < 2) was used to extrapolate biomass.

To evaluate the relationship between MNA and vegetation variables, we constructed log‐linked Poisson generalized linear mixed‐effect models (GLMMs). We used MNA as the response variable, seven habitat variables as explanatory variables (ro, mo, lich, dl, shr, avshr, and biomass [biom]), the grid location (site among the six sites listed under “study area” above) as fixed effect, and grid as a random factor. To evaluate whether such a relationship may have a delayed effect on MNA, various data sets were created by shifting the abundances with respect to the explanatory variables. Each sampling session where vegetation data were available was assigned the MNA measured a given number of months later (lag). Incomplete entries were discarded. For each lag between 0 and 16 months, GLMMs including all the variables and all nested models were fitted and averaged using AIC weights with a correction for finite sample sizes (AICc). Model fitting and averaging were conducted using R packages lme4 (Bolker, 2013) and MuMIn (Barton, 2013), respectively. Only models with ΔAICc < 10 were included in the average (Burnham & Anderson, 2002). No assessment of significance other than model selection was made at this stage.

To account for the error in the extrapolation of biomass, the analysis described above was repeated following a randomization procedure. For each replicate, a new random variable was generated for each entry for which biomass was extrapolated, drawing its value from a normal distribution with the mean equal to the extrapolated value and standard deviation equal to the extrapolation error. The randomization‐fitting cycle was repeated 350 times. This number of replicates was decided upon a preliminary analysis, so that the standard error of averaged coefficients would be smaller than their corresponding errors in each replicate. Final errors in the replicate‐averaged model were calculated considering single‐replicate errors and the dispersion due to randomizing extrapolated biomass. The error of each coefficient α was estimated as

$$E_{\alpha }=\sqrt{\frac{1}{N}\sum_{i=1}^{N}\frac{(\bar{\alpha }-{\alpha_{i})}^{2}}{N-1}+SE{\left(\alpha_{i}\right)}^{2}},$$

where α_*i*_ are the estimated values from replicate *i* (1 < *i* < *N*), $\bar{\alpha }=\frac{1}{N}\sum_{i=1}^{N}\alpha_{i}$ is their mean value, and *SE*(α_*i*_) is their standard error from each replicate. Effects for a given time lag were considered significant when the corresponding 95% confidence intervals ($\bar{\alpha }\pm 1.96E_{\alpha }$) did not include zero. In order to test for possible density‐dependent effects, we investigated the combined effects of previous abundances and lagged values of biomass on deer mouse populations. The use of autoregressive models (i.e., models for which each observation of the response variable is modeled in terms of other observations of the same variable) has proved useful for understanding important correlations—both temporal and spatial—in ecology (Vieira et al. 2008, Ives et al. 2010). Applying these models to the present data is not straightforward as trapping sessions were not always evenly spaced. However, trapping sessions were conducted often enough so that characteristic times of population dynamics comprised multiple sessions. Therefore, we adopted a *coarser* approach: For each sampling session, we calculated the log‐transformed (i.e., log[1 + *x*]) mean abundances of three previous 6‐month periods (short term: 1–6 months, midterm: 7–12 months, and long term: 13–18 months prior to current session). These three averages, sampling grid, and the mean biomass for the three time lags which showed stronger effects (8–10 months prior to current session, see 3) were considered as covariates in a log‐linked Poisson GLM. Two‐ and threefold interaction terms among biomass, previous abundances, and site were also included in the full model (but no interactions among averaged abundances). We grouped interactions per site (instead of per grid) to avoid having too many parameters to estimate. The full model and all nested models were fitted and averaged based on their AICc (Burnham & Anderson, 2002), using package MuMIn for R (Barton, 2013). Relative importance (RI) of each term was calculated as the sum of Akaike weights of all models having that term. For this analysis, data from site Wisdom were excluded due to consistently too low capture rates.

## 3 RESULTS

**Figure 1:**
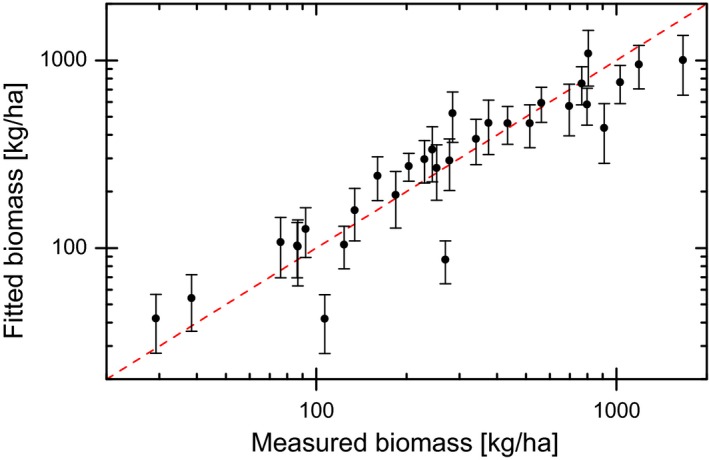
Relationship of fitted (from plant cover data) to measured values of biomass in Montana. Error bars enclose the 95% confidence interval (mean ± 1.96 *SE*). The red dashed line represents a 1:1 relationship and is included as a visual guide

Measured biomass ranged from 29 to 1,666 kg/ha. Fitted biomass was in agreement with measured values, within estimated errors (Figure 1). Observed MNA ranged from 0 to 170 individuals per trapping grid. Datasets obtained after shifting MNA with respect to habitat variables comprised between 55 and 348 entries for each time lag. For most time lags, the standard deviation of all coefficients through replicates was similar to individual replicate errors, indicating that biomass extrapolation errors had little impact. Moreover, standard errors of averaged coefficients were smaller than individual replicate errors, supporting the robustness of our replication procedure.

**Figure 2:**
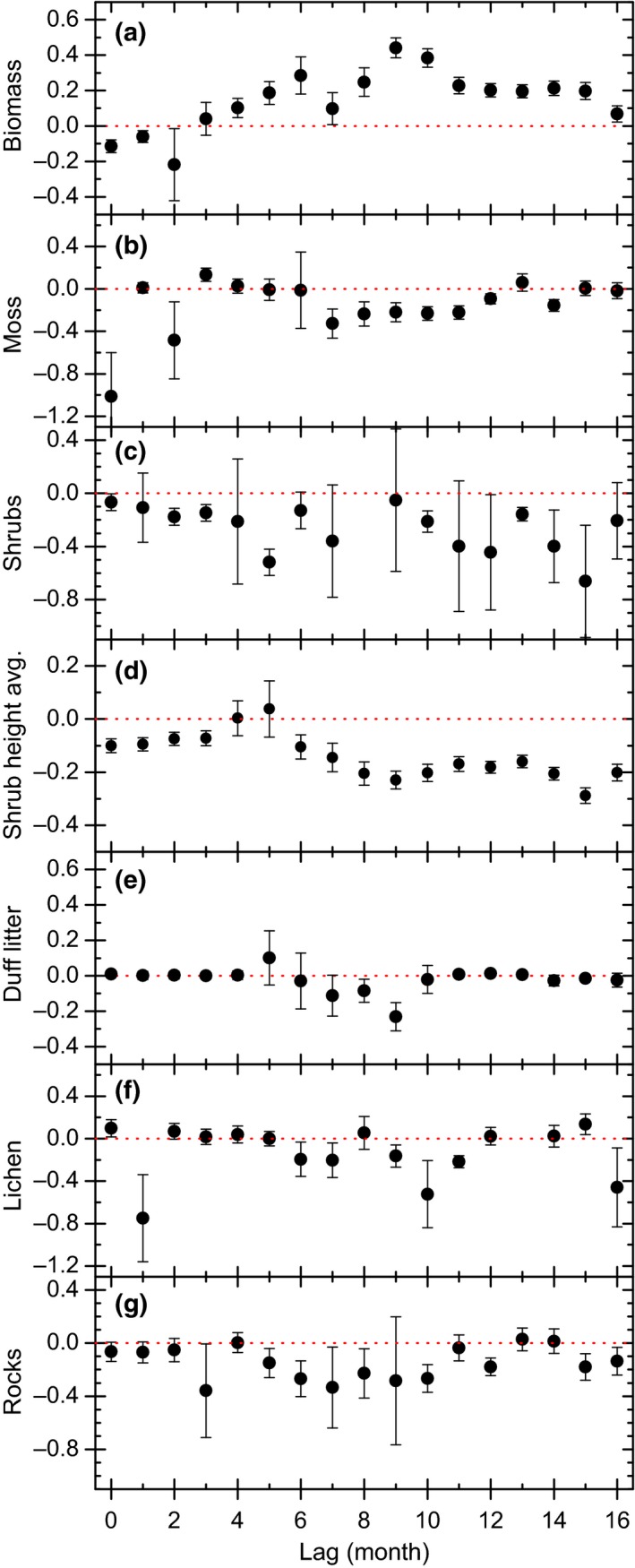
Generalized linear mixed‐effect models regression coefficients for vegetation covariates associated with minimum number alive for various time lags averaged over 350 randomized replicates. Error bars enclose the 95% confidence interval (mean ± 1.96 *SE*). Each panel (labeled a‐g) corresponds to a different variable, indicated by the y‐axis title

Averaged coefficients of fitted GLMMs, corresponding to each habitat variable and for each time lag, are shown in Figure 2 with their respective estimated errors. There was a positive relationship between MNA and biomass (Figure 2a) for lags ≥4 months, with a maximum effect at 9‐ and 10‐month lags. The negative effect of biomass on MNA observed for short lags (<3 months) may be an artifact due to temporal self‐correlation. Additionally, MNA was negatively related with shrub cover (Figure 2c) or residuals of the average shrub height (Figure 2d) for every time lag, except for a 4‐month lag. The fact that shrub cover and the residuals of average shrub height had a negative correlation with MNA for most time lags indicates a constant effect. Moreover, the residuals of average shrub height displayed more consistent association than shrub cover per se, suggesting that deer mice were less abundant in places with tall shrubs. The remaining habitat variables displayed a negative effect for some time lags: 0, 2, 7–12, and 14 months for moss (Figure 2b); 7–9 months for duff litter (Figure 2e); 1, 6, 7, and 9–11 months for lichens (Figure 2f); and 5–8, 10, 12, 15, and 16 months for rocks (Figure 2g). Slight positive effects of moss and lichens on MNA were observed for 3‐month and 0‐month lags, respectively.

**Figure 3:**
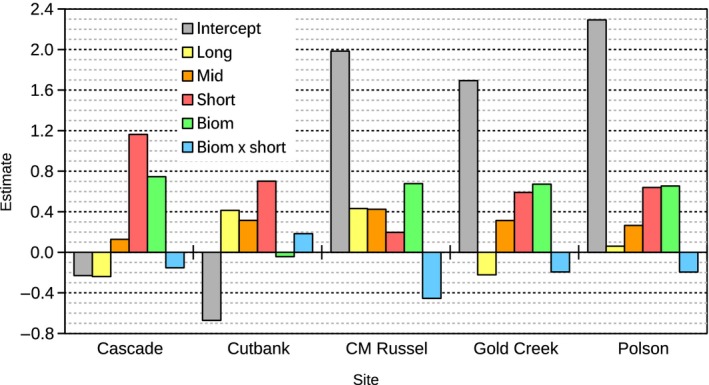
Per‐site averaged net effects of previous abundances and lagged values of biomass, estimated from autoregressive models. Only coefficients for terms with stronger support in the averaged model (RI ≥ 0.98) are shown

**Figure 4:**
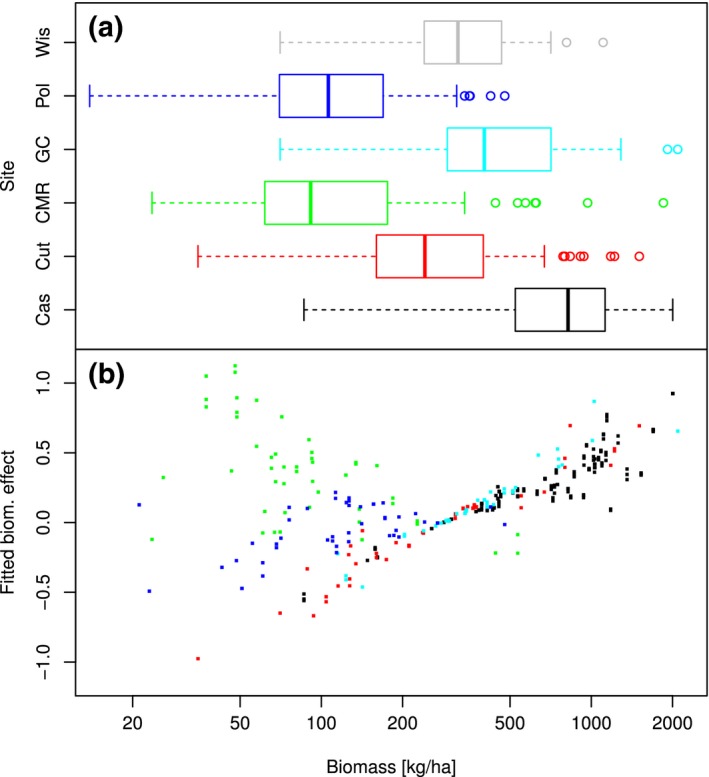
(a) Boxplot representation of measured and extrapolated biomass values distributions per site (Cas = Cascade, Cut = Cutbank, CMR = C.M. Russell wildlife refuge, GC = Gold Creek, Pol = Polson, and Wis = Wisdom). (b) Contribution of biomass to the linear predictor of minimum number alive in the averaged GLM, per site. Color of each data series matches the corresponding site in panel (a)

The analysis of density‐dependent effects was conducted on a dataset of 329 entries, for which it was possible to properly calculate the required averaged lagged values. Figure 3 shows the averaged coefficients for terms with stronger support in the averaged model (RI ≥ 0.98, whereas for other terms RI < 0.6). Here, site interactions were added to main terms in order to display net effects at each site. Short‐ and midterm averaged previous MNAs were positively associated with current MNA at all sites, indicating that all populations were rather stable (positively correlated) at these timescales. This is not surprising as typical life spans of deer mice (between 1 and 2.5 years in the wild, ref.) are longer than these periods. In contrast, long‐term averaged previous MNAs were not uniformly related with current MNA across five sites: Coefficient estimates were positive in Cutbank, CMR, and Polson (albeit almost null here), but negative in Cascade and Gold Creek. This difference may imply that population dynamics are slower in the former than in the latter. For all sites except Cutbank, lagged biomass had a positive effect on current MNA and displayed a negative interaction with short‐term averaged previous MNA. The strength of the main effect was similar across the four sites, whereas the negative interaction was particularly stronger at CMR. Results for Cutbank—second lowest in densities after Wisdom, the excluded site—show a weak negative main effect of biomass and a positive interaction with short‐term averaged previous MNA. However, in order to properly assess the effects of biomass, the interaction term has to be weighed by the corresponding covariate. Figure 4 shows the range of biomass values (both measured and extrapolated) for each site and the fitted effect of biomass. The latter was calculated as

$$\bar{\text{biom}}\times \left[\alpha_{\mathrm{biom}}+\alpha_{\mathrm{inter}}\times \text{log}\left(1+\bar{\text{MNA}}\right)\right],$$

where $\bar{\text{biom}}$ is the averaged lagged biomass, α_biom_ and α_inter_ are the main and interaction coefficients for biomass, respectively, and $\bar{\text{MNA}}$ is the short‐term averaged previous MNA. In the three sites with higher biomass (Cascade, Cutbank, and Gold Creek), abundance exhibited a similar positive response to biomass, which becomes less steep for higher biomass values. In contrast, in the other two (CMR and Polson), main and interaction terms cancel each other, and no consistent effect of biomass is evident.

## 4 DISCUSSION

The bottom‐up regulation model assumes that energy (i.e., food) is the only factor limiting populations, so their densities should increase continuously with greater food availability. Plant material (mostly as seeds but some vegetative parts) directly provides energy and supports many insect populations which are also a source of energy for deer mouse populations (Pearson & Callaway, 2006). Therefore, rodent populations should expand after periods of warm temperatures and abundant precipitation due to the subsequent increase in plant productivity (Hansson, 1979). Considering all the stages in a bottom‐up model, the maximum positive effect on rodent abundance can be expected about a year after warm and rainy weather for several reasons (Heisler, Somers, & Poulin, 2014): It takes a growing season between precipitation and the expression of productivity. Once mice receive adequate biomass, it takes time for the population to respond through survival and reproduction. The same applies for insect populations before they represent an increased source of energy for mice.

When the conditions for a bottom‐up regulation are met, a sudden increase in food availability may unleash a population explosion. Examples of such situations outside the US southwest are found worldwide: In temperate Europe, bank voles populations increase after mast years (Johnson, Moraes Figueiredo, & Vapalahti, 2010); in western Patagonia, infrequent flowering of colihue cane is followed by a drastic growth of granivorous rodent populations (Jaksic & Lima, 2003; Piudo, Monteverde, González Capria, Padula, & Carmanch, 2004). However, the same species subject to bottom‐up regulation in one region may show completely different dynamics in another context, such as the contrasting predation‐driven top‐down regulation of bank voles in northern Europe (Johnson et al., 2010).

While there is sound evidence of a precipitation‐driven bottom‐up process ruling deer mouse population dynamics in the arid southwestern United States and the Channel Islands in California (Orrock et al., 2011), the mechanism is not so clear in the western mesic habitats. Below, we analyze whether the conditions for a precipitation‐driven bottom‐up regulation are met in Montana study sites.

### 4.1 Food as the only limiting factor

Liebig's law of the minimum states that population growth will always be controlled by the scarcest essential resource (Salisbury, 1992). In this context, energy must be the only limiting factor. Other requirements such as nest sites and escape cover cannot be more limiting than energy. Food effects have been tested with several species of rodents. An increase in food results in various demographic changes (Duquette & Millar, 1995), but neither increased food (Duquette & Millar, 1995; Wolff, 1985) nor natural seed production (Kaufman et al. 1995; Elkington et al., 1996) necessarily increased population density. Increased mast yield does increase deer mouse population density (Ostfeld, Jones, & Wolff, 1996; Wolff, 1985; Schnurr et al. 2002), but there were no mast‐producing plants on our study sites. Thus, it is doubtful that energy is always the only limiting factor for our study populations.

The average response across six sampling locations to an increase in plant productivity was delayed increased MNA. The maximum positive effect of biomass on deer mouse abundance occurred after a 9‐month lag. This suggests a connection between plant productivity and rodent population growth. However, the lagged effect of biomass on MNA was relevant only in Cascade, Cutbank, and Gold Creek, the three locations with higher plant productivity in this study. Lack of an association between biomass and abundance at Polson and C. M. Russell sites indicates that plant productivity, albeit low, is not a limiting factor for local populations. On the contrary, deer mice were scarce in Wisdom site, although biomass values were typically higher than in Polson and C. M. Russell sites. While the low abundances in Wisdom are likely due to high elevation of this site, it is not clear why deer mouse populations in Cascade, Cutbank, and Gold Creek are so strongly dependent on plant productivity whereas those in Polson and C. M. Russell are sustained with so little available biomass. It is possible that the latter relied on a different source of food, not accounted by the measured biomass.

Our finding that directly measured productivity was a good predictor of population growth in Cascade is contrasting with previous work by Loehman et al. (2012), who found no correlation between deer mouse abundance and remotely sensed plant productivity in Cascade and Polson study sites. This may indicate that the large size of the remotely sensed area in Loehman et al.'s work may not accurately account for smaller‐scale patterns which drive population dynamics locally.

Other habitat features, including rocks, duff litter, moss, and lichens, had a negative association with abundance only for some time lags, mostly around 7–10 months. This is coincident with the strongest positive association with biomass, suggesting that there is a connection among all effects. Rocky environments affect nesting habitats (Wolff & Sherman, 2008) while moss and lichens may be indicators of recent climatic conditions such as humidity and temperature or habitat quality. It is possible that these abiotic factors also affected deer mouse survival, and due to the characteristic times of their population dynamics, all the effects on MNA become apparent after about the same time lag.

### 4.2 Density‐independent behavior

In order for populations to grow as long as additional food becomes available, intraspecific interactions must remain constant through all population densities. If deer mice were territorial, their numbers may be limited by social behavior before resources become limiting (Krebs et al., 1973).

Previous work by Lonner, Douglass, Kuenzi, and Hughes (2008) and Waltee, Lonner, Kuenzi, and Douglass (2009) reported the effects of population density on dispersal at Cascade and Polson study sites in Montana, where they found that dispersal increased as population density increased. Fairbairn (1978) reported similar behavior of *P. maniculatus* in Vancouver, Canada. This indicates that at least the phenotypic behavior of deer mice changes with population density, in that mice became intolerant of each other (territorial) and some left the area. Further evidence of territoriality occurred during a peridomestic study by Douglass, Kuenzi, Williams, Douglass, and Mills (2003), when deer mice removed from buildings were quickly replaced by new mice. In control buildings, mouse populations remained stable and were comprised of the same individuals for the duration of the study.

Our results offer evidence that such territoriality may indeed constrain the effects of forage (biomass). For Cascade and Gold Creek study sites (both with highest abundances out of the three sites where we found a positive effect of biomass on MNA), the interaction term between lagged biomass and short‐term previous MNA was negative. This appears as a slight saturation in the fitted effect of lagged biomass on MNA (Figure 4). The meaning of this saturation is that population growth resulting from increased plant productivity becomes less pronounced in moments of higher abundance, thus supporting the existence of a density‐dependent social limit.

### 4.3 No interspecific competition or predation

Competing species may interfere with deer mice using available energy, thus reducing the impact of changes in plant productivity on mice populations. The most abundant other small‐mammal species at the study sites were voles (*Microtus* sp.), which were only present sporadically on the grids. Small‐mammal communities at our study sites were relatively simple compared to studies conducted in the US southwest (Douglass & Vadell, 2016). Therefore, although competition (either by aggressive interference or by simply getting to the food first) may have occurred at some point on some of our grids, we can expect that it was not a strong factor determining deer mouse abundance at our study sites.

On the other hand, increased survival or recruitment as a consequence of increased food availability could be countered by increased predation, leading to a mixture of top‐down and bottom‐up processes (Prevedello et al., 2013). The predators' coyote (*Canis latrans*), ermine (*Mustela erminia*), and rattlesnake (*Crotalus viridus*) were occasionally observed or trapped on or near various grids. We do not have data on the effect of these predators on deer mouse abundance in our study sites. However, in the Channel Islands in California, predator richness has been associated with lower hantavirus prevalence, likely as result of reduced deer mouse density (Orrock et al., 2011). Kotler (1984) documented predation on deer mice by owls in the Great Basin Desert. Later, Kotler (1985) described avoidance of open areas and foraging in bushes as antipredation strategies, which eventually determined microhabitat use. Reduced foraging activity of deer mice in response to artificial light was also observed in experiments (Clarke 1983). If density‐dependent behavior forces some individuals to forage in open areas due to increased density, predation risk also increases at higher densities. Thus, predation may limit population growth as a consequence of the social limit caused by intraspecific strife. However, this compensatory effect is expected to be secondary to that of plant productivity (Mutshinda, O'Hara, & Woiwod, 2009; Ostfeld & Holt, 2004).

### 4.4 Only productivity‐mediated effects of precipitation affect deer mouse populations

In the precipitation‐driven bottom‐up model, precipitation effects on populations are mediated by plant productivity. Therefore, it is an indirect effect which should become apparent only several months after precipitation occurred. Plant productivity at the relatively dry Montana sites (typical annual precipitation <35 cm) increases with greater precipitation. Luis et al. (2010) showed that higher temperature and more precipitation during summer through early winter were important in determining deer mouse survival after a 5‐month lag in Cascade study site. Our finding of a delayed positive effect of biomass on MNA in Cascade and two other study sites, together with Luis et al.' observations, indicates that rain would have positive effects on deer mouse survival through improved plant productivity in these locations.

However, precipitation was also present in the form of snow cover and duration, almost every winter and sometimes in May and September throughout the duration of the study. While snow supplied significant water and likely increased plant productivity, it is not clear whether it may have also had a direct effect on populations. In northern Europe, snow has been found to provide shelter, reducing predation risks during winter (Hansson 1985). In contrast, Douglass and Vadell (2016) reported populations reached annual lows on all grids at the end of winter, with the exception of mild winters (no midwinter snow accumulation) when deer mouse population numbers were higher. This suggests a negative relationship between snow accumulation and overwinter survival. It is not clear whether this is actually a consequence of snow or it is due to more general weather conditions correlated with snow accumulation (e.g., lower temperatures). In either case, should plant productivity be increased after snowy winters, reduced overwinter survival will limit the benefits of subsequently increased food availability.

### 4.5 A population regulation model for deer mouse populations in the Northern Great Plains

**Figure 5:**
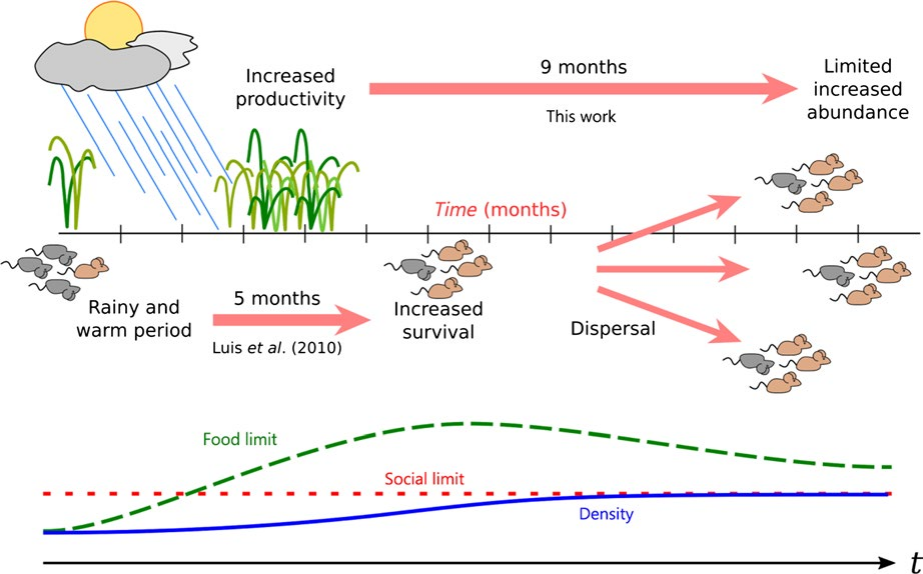
Schematic description of the damped trophic cascade timeline in Montana (green dashed line represents the population density theoretically allowed by the food supply alone; red dotted line (social limit) is the population density allowed by density‐dependent factors). In periods of low food availability, rodent survival (represented by brown/gray mice ratio) may be limited by food. After warm and rainy periods which increase plant productivity, higher food availability may enhance survival, leading to population growth. However, once the population density (blue line) approaches a social limit, mice disperse despite surplus food availability. Local abundance thus increases, but not as much as expected if food were the only limiting factor

Periods of low food availability acting as a limiting factor for deer mouse were observed at three study sites, but population fluctuations at two other sites could not be explained in terms of biomass availability. Moreover, at the three study sites where biomass was related to increased MNA, the food limit would not be much lower than the social limit due to intraspecific competition and density‐dependent behavior. Therefore, increased food availability likely enhances survival and leads to population growth, but individuals soon leave crowded areas. Dispersal thus attenuates the local density increment below the higher food limit (Figure 5).

Increased predation may occur as a result of higher rodent density, but most likely after abundance is already limited by density‐dependent interactions. For this reason, this increment in predation would not be a crossover from bottom‐up to top‐down regulation as more prey becomes available for predators (Orrock et al., 2011; Prevedello et al., 2013). Instead, populations are limited by a combination of energy availability and social behavior, leading to a damped bottom‐up process. Other abiotic factors, such as snow accumulation and availability of nesting sites, may also contribute to compensate beneficial effects of increased plant productivity in response to precipitation.

Still, the proposed damped precipitation‐driven bottom‐up model adequately explains the observed dynamics only in the three study sites where consistent fluctuations in response to measured biomass were observed. What energy source replaces biomass at the study sites with lower measured plant productivity and whether it acts as a limiting factor remain to be explored.

## 5 CONCLUSION

In the arid US southwest, deer mouse populations expand after El Niño events that produce widespread plant growth where typically little growth occurs during dry years (Parmenter et al., 1993; Yates et al., 2002). Similar strong associations between precipitation and rodent density, mediated by increased plant productivity, were observed in the Channel Islands in California (Orrock et al., 2011). In contrast, the conditions required for a strictly precipitation‐driven bottom‐up regulation to occur are only partly met by persistent deer mouse populations in Montana. Although we found positive correlations between deer mouse abundance and plant productivity with a several‐month lag, as required to fit the hypothesized upward trophic cascade model, the effect was neither particularly strong nor universal over 18 livetrapping grids in Montana. Predation and interspecific competition appear to be of little importance in regulating deer mouse populations in Montana study sites, but social and abiotic factors may play roles not observed in desert habitats of the US southwest.

It is clear that deer mouse populations in northwestern Montana display complex dynamics which requires consideration of multiple potential limiting factors (Heisler et al., 2014). Thus, a combination of factors prevents dramatic changes in rodent abundances after sudden increments of food availability, such as those observed in other regions.

## AUTHOR CONTRIBUTIONS

Irene L. Gorosito performed all of the data analysis, participated in field sampling during one season, and contributed significantly to the manuscript preparation. Richard J. Douglass secured funding for deer mouse/SNV research in Montana from CDC, NIH, and other agencies. Douglass designed, participated in fieldwork, supervised all aspects of the study, and contributed to preparation of the manuscript.

## CONFLICT OF INTEREST

None declared.

## References

[ece33598-bib-0001] Barton, K. (2013). MuMIn: Model selection and model averaging based on information criteria. R package version 1.9.5. Retrieved from http//CRAN.R-project.org/package=MuMIn.

[ece33598-bib-0002] Bolker, B. M. (2013). lme4: Linear mixed‐effects models using Eigen and S4. R package version 1.0‐4. Retrieved from http//CRAN.R-project.org/package=lme4

[ece33598-bib-0003] Burnham, K. P. (1983). The jackknife, the bootstrap and other resampling plans. by B. Efron. Biometrics, 39, 816–817. https://doi.org/10.2307/2531123

[ece33598-bib-0004] Burnham, K. P. , & Anderson, D. R. (2002). Model selection and multimodel inference: A practical information‐theoretic approach, 2nd ed New York, NY, USA: Springer‐Verlag.

[ece33598-bib-0005] Carver, S. , Mills, J.N. , Parmenter, C.A. , Parmenter, R.R. , Richardson, K.S. , Harris, R.L. , … Luis, A.D. (2015). Toward a mechanistic understanding of environmentally forced zoonotic disease emergence: Sin Nombre Hantavirus. Bio Science, 65, 651–666. https://doi.org/10.1093/biosci/biv047 10.1093/biosci/biv047 PMC4776718 26955081

[ece33598-bib-0006] Chitty, D. , & Phipps, E. (1966). Seasonal changes in survival in mixed populations of two species of vole. Journal of Animal Ecology, 35, 313–331. https://doi.org/10.2307/2398

[ece33598-bib-0007] Christian, J. J. (1950). The adreno‐pituitary system and population cycles in mammals. Journal of Mammalogy, 31, 247–259. https://doi.org/10.2307/1375290

[ece33598-bib-0008] Christian, J. J. (1978). Neurobehavioral endocrine regulation of small mammal populations In Snyder D. P. (Ed.), Populations of small mammals under natural conditions, Vol. 5 (pp. 143–158). Linesville, PA, USA: Special Publication Series, Pymatuning Laboratory of Ecology, University of Pittsburgh.

[ece33598-bib-0501] Clarke, J. A. (1983). Moonlights influence on predator/prey interactions between short‐eared owls (Asio flammeus) and deermice (Peromyscus maniculatus). Behavioral Ecology and Sociobiology, 13, 205–209. https://doi.org/10.1007%2fbf00299924

[ece33598-bib-0009] Conley, W. (1976). Competition between Microtus: A behavioral hypothesis. Ecology, 57, 224–237. https://doi.org/10.2307/1934812

[ece33598-bib-0010] David, E. D. (1978). Physiological and behavioral responses to the social environment In Snyder D. P. (Ed.), Populations of small mammals under natural conditions, Vol. 5 (pp. 84–91). Linesville, PA, USA: Special Publication Series, Pymatuning Laboratory of Ecology, University of Pittsburgh.

[ece33598-bib-0011] Dearing, M. D. , & Dizney, L. (2010). Ecology of Hantavirus in a changing world. New York Academy of Sciences, 1195, 99–112. https://doi.org/10.1111/j.1749-6632.2010.05452.x 10.1111/j.1749-6632.2010.05452.x 20536819

[ece33598-bib-0012] Douglass, R. J. , & Vadell, M. V. (2016). How much effort is required to accurately describe the complex ecology of a rodent‐borne viral disease? Ecosphere, 7, e01368 https://doi.org/10.1002/ecs2.1368 27398256 10.1002/ecs2.1368 PMC4930245

[ece33598-bib-0013] Douglass, R. J. , Kuenzi, A. J. , Williams, C. Y. , Douglass, S. J. , & Mills, J. N. (2003). Removing deer mice from buildings: Potential effects on risk of human exposure to Sin Nombre virus. Emerging Infectious Diseases, 9, 390–392. https://doi.org/10.3201/eid0903.020470 12643840 10.3201/eid0903.020470 PMC2958549

[ece33598-bib-0014] Douglass, R. J. , Kuenzi, A. J. , Wilson, T. , & Van Horne, R. C. (2000). Effects of bleeding non‐anesthetized wild rodents on handling mortality and subsequent recapture. Journal of Wildlife Diseases, 36, 700–704. https://doi.org/10.7589/0090-3558-36.4.700 11085431 10.7589/0090-3558-36.4.700

[ece33598-bib-0015] Douglass, R. J. , & Vadell, M. V. (2016). How much effort is required to accurately describe the complex ecology of a rodent‐borne viral disease? Ecosphere, 7, 1–8. 10.1002/ecs2.1368 PMC4930245 27398256

[ece33598-bib-0016] Douglass, R. J. , Van Horn, R. , Coffin, K. W. , & Zanto, S. N. (1996). Hantavirus in Montana deer mouse populations: Preliminary results. Journal of Wildlife Diseases, 32, 527–530. https://doi.org/10.7589/0090-3558-32.3.527 8827681 10.7589/0090-3558-32.3.527

[ece33598-bib-0017] Douglass, R. J. , Wilson, T. , Semmens, W. J. , Zanto, S. N. , Bond, C. W. , Van Horn, R. C. , & Mills, J. N. (2001). Longitudinal studies of Sin Nombre virus in deer mouse‐dominated ecosystems of Montana. American Journal of Tropical Medicine and Hygiene, 65, 33–41. https://doi.org/10.4269/ajtmh.2001.65.33 11504405 10.4269/ajtmh.2001.65.33

[ece33598-bib-0018] Duquette, L. S. , & Millar, J. S. (1995). The effect of supplemental food on life‐history traits and demography of a tropical mouse *Peromyscus mexicanus* . Journal of Animal Ecology, 64, 348–360. https://doi.org/10.2307/5896

[ece33598-bib-0019] Elkington, J. S. , Healy, W. M. , Buonaccorsi, J. P. , Boettner, G. H. , Hazzard, A. M. , Smith, H. R. , & Liebhold, A. M. (1996). Interactions among gipsy moths, white‐footed mice and acorns. Ecology, 77, 2332–2342. https://doi.org/10.2307/2265735

[ece33598-bib-0020] Engelthaler, D. M. (1999). Climatic and environmental patterns associated with hantavirus pulmonary syndrome, Four Corners region, United States. Emerging Infectious Diseases, 5, 87–94. https://doi.org/10.3201/eid0501.990110 10081675 10.3201/eid0501.990110 PMC2627709

[ece33598-bib-0502] Faribairn, D. J. (1978). Dispersal of deer mice, *Peromyscus maniculatus*, proximal causes and effects on fitness. Oecologia, 32, 171–193. 28309396 10.1007/BF00366070

[ece33598-bib-0503] Glass, G. E. , Cheek, J. E. , Patz, J. A. , Shields, T. M. , Doyle, T. J. , Thoroughman, D. A. , … Bryan, R. (2000). Using remotely sensed data to identify areas at risk for hantavirus pulmonary syndrome. Emerging Infectious Diseases, 6, 238–247. https://doi.org/10.3201/eid0603.000303 10827113 10.3201/eid0603.000303 PMC2640870

[ece33598-bib-0504] Glass, G. E. , Cheek, J. E. , Patz, J. A. , Shields, T. M. , Doyle, T. J. , Thoroughman, D. A. , … Bryan, R. (2000). Using remotely sensed data to identify areas at risk for hantavirus pulmonary syndrome. Emerging Infectious Diseases, 6, 238–247. 10827113 10.3201/eid0603.000303 PMC2640870

[ece33598-bib-0021] Glass, G. E. , Cheek, J. E. , Patz, J. A. , Shields, T. M. , Doyle, T. J. , Thoroughman, D. A. , … Mills, J. N. (2002). Satellite imagery characterizes local animal reservoir populations of Sin Nombre virus in the southwestern United States. Proceedings of the National Academy of Sciences of the United States of America, 99, 16817–16822. https://doi.org/10.1073/pnas.252617999 12473747 10.1073/pnas.252617999 PMC139227

[ece33598-bib-0022] Glass, G. E. , Shields, T. , Cai, B. , Yates, T. L. , & Parmenter, R. (2007). Persistently highest risk areas for hantavirus pulmonary syndrome: Potential sites for refugia. Ecological Applications, 17, 129–139. https://doi.org/10.1890/1051-0761(2007)017[0129:PHRAFH]2.0.CO;2 17479840 10.1890/1051-0761(2007)017[0129:phrafh]2.0.co;2

[ece33598-bib-0023] Glass, G. E. , Shields, T. , Parmenter, R. R. , Goade, D. , Mills, J. N. , Cheek, J. , … Yates, T. L. (2006). Predicted Hantavirus risk in 2006 for the southwestern US (p. 255). Occasional Paper no: Museum of Texas Tech University.

[ece33598-bib-0505] Goodwin, L. D. , & Leech, N. L. (2006). Understanding correlation: Factors that affect the size of r. The Journal of Experimental Education, 74, 249–266. https://doi.org/10.3200/jexe.74.3.249-266

[ece33598-bib-0024] Hansson, L. (1979). Food as a limiting factor for small rodent numbers: Test of two hypotheses. Oecologia, 37, 297–314. https://doi.org/10.1007/BF00347907 28309217 10.1007/BF00347907

[ece33598-bib-0506] Hansson, L. , & Henttonen, H. (1985). Gradients in density variations of small rodents: The importance of latitude and snow. Oecologia, 67, 394–402. http://www.jstor.org/stable/4217749. 28311574 10.1007/BF00384946

[ece33598-bib-0025] Heisler, L. M. , Somers, C. M. , & Poulin, R. G. (2014). Rodent populations on the northern Great Plains respond to weather variation at a landscape scale. Journal of Mammalogy, 95, 82–90. https://doi.org/10.1644/13-MAMM-A-115.1

[ece33598-bib-0026] Hunt, D. K. , Enscore, R. E. , Gage, K. L. , Irland, C. , Peters, C. J. , & Bryan, R. (2000). Using remotely sensed data to identify areas at risk for Hantavirus pulmonary syndrome. Emerging Infectious Diseases, 6, 238–247. 10827113 10.3201/eid0603.000303 PMC2640870

[ece33598-bib-0507] Ives, A. R. , Abbott, K. C. , & Ziebarth, N. L. (2010). Analysis of ecological time series with ARMA(p, q) models. Ecology, 9, 858–871. https://doi.org/10.1890/09-0442.1 10.1890/09-0442.1 20426343

[ece33598-bib-0027] Jaksic, F. M. , & Lima, M. (2003). Myths and facts on ratadas: Bamboo blooms, rainfall peaks and rodent outbreaks in South America. Austral Ecology, 28, 237–251. https://doi.org/10.1046/j.1442-9993.2003.01271.x

[ece33598-bib-0028] Johnson, C. B. , Moraes Figueiredo, L. T. , & Vapalahti, O. (2010). A global perspective on Hantavirus ecology, epidemiology, and disease. Clinical Microbiology Reviews, 23, 412–441. https://doi.org/10.1128/CMR.00062-09 20375360 10.1128/CMR.00062-09 PMC2863364

[ece33598-bib-0508] Kaufman, D. M. (1995). Diversity of new world mammals: Universality of the latitudinal gradients of species and Bauplans. Journal of Mammalogy, 76, 322–334. https://doi.org/10.2307/1382344

[ece33598-bib-0029] Kotler, B. P. (1984). Harvesting rates and predatory risk in desert rodents: A comparison of two communities on different continents. Journal of Mammalogy, 65, 91–96. https://doi.org/10.2307/1381204

[ece33598-bib-0030] Kotler, B. P. (1985). Owl predation on desert rodents which differ in morphology and behavior. Journal of Mammalogy, 66, 824–828. https://doi.org/10.2307/1380824

[ece33598-bib-0031] Krebs, C. J. , Gaines, M. S. , Keller, B. L. , Myers, J. H. , & Tamarin, R. H. (1973). Population cycles in small rodents. Science, 179, 35–41. https://doi.org/10.1126/science.179.4068.35 4734149 10.1126/science.179.4068.35

[ece33598-bib-0032] Lack, D. (1954). Cyclic mortality. The Journal of Wildlife Management, 18, 25–37. https://doi.org/10.2307/3797612

[ece33598-bib-0033] Loehman, R. A. , Ellas, J. , Douglass, R. J. , Kuenzi, A. J. , Mills, J. N. , & Wagoner, K. (2012). Prediction of *Peromyscus maniculatus* (deer mouse) population dynamics in Montana, USA, using satellite driven vegetation productivity and weather data. Journal of Wildlife Diseases, 48, 348–360. https://doi.org/10.7589/0090-3558-48.2.348 22493110 10.7589/0090-3558-48.2.348 PMC3572777

[ece33598-bib-0034] Lonner, B. N. , Douglass, R. J. , Kuenzi, A. J. , & Hughes, K. (2008). Seroprevalence against Sin Nombre virus in resident and dispersing deer mice. Vector Borne and Zoonotic Diseases, 8, 433–442. https://doi.org/10.1089/vbz.2007.0232 18447620 10.1089/vbz.2007.0232 PMC2978542

[ece33598-bib-0035] Luis, A. D. , Douglass, R. J. , Mills, J. N. , & Bjørnstad, O. N. (2010). The effect of seasonality, density and climate on the population dynamics of Montana deer mice, important reservoir hosts for Sin Nombre hantavirus. Journal of Animal Ecology, 79, 462–470. https://doi.org/10.1111/jae.2010.79.issue-2 20015212 10.1111/j.1365-2656.2009.01646.x

[ece33598-bib-0037] Mills, J. N. (2005) Regulation of Rodent‐Borne viruses in the natural host: Implications for human disease In Peters C. J. & Calisher C. H. (Eds.), Infectious diseases from nature: Mechanisms of viral emergence and persistence (pp. 45–57). Vienna: Springer https://doi.org/10.1007/3-211-29981-5_5 10.1007/3-211-29981-5_5 16355867

[ece33598-bib-0038] Mills, J. N. , Ksiazek, T. G. , Peters, C. J. , & Childs, J. E. (1999). Long‐term studies of hantavirus reservoir populations in the southwestern United States: A synthesis. Emerging Infectious Diseases, 5, 135–142. https://doi.org/10.3201/eid0501.990116 10081681 10.3201/eid0501.990116 PMC2627702

[ece33598-bib-0039] Mutshinda, C. M. , O'Hara, R. B. , & Woiwod, I. P. (2009). What drives community dynamics? Proceedings of the Royal Society B, 276, 2923–2929. https://doi.org/10.1098/rspb.2009.0523 19457887 10.1098/rspb.2009.0523 PMC2817208

[ece33598-bib-0040] Myers, J. H. , & Krebs, C. J. (1971). Genetic, behavioral, and reproductive attributes of dispersing field voles Microtus pennsylvanicus and Microtus ochrogaster. Ecological Monographs, 41, 53–78. https://doi.org/10.2307/1942435

[ece33598-bib-0041] Orrock, J. L. , Allan, B. F. , & Drost, C. A. (2011). Biogeographic and ecological regulation of disease: Prevalence of Sin Nombre virus in Island mice is related to Island Area, precipitation, and predator richness. The American Naturalist, 177, 691–697. https://doi.org/10.1086/659632 10.1086/659632 21508614

[ece33598-bib-0042] Ostfeld, R. S. , & Holt, R. D. (2004). Are predators good for your health? Evaluating evidence for top‐down regulation of zoonotic disease reservoirs. Frontiers in Ecology and Environment, 2, 13–20. https://doi.org/10.1890/1540-9295(2004)002[0013:APGFYH]2.0.CO;2

[ece33598-bib-0043] Ostfeld, R. S. , Jones, C. G. , & Wolff, J. O. (1996). Of mice and mast, Ecological connections in eastern deciduous forest. BioScience, 46, 323–330. https://doi.org/10.2307/1312946

[ece33598-bib-0044] Parmenter, R. R. , Brunt, J. W. , Moore, D. I. , & Ernest, S. (1993). The Hantavirus epidemic in the Southwest: Rodent population dynamics and the implications for transmission of hantavirus‐associated adult respiratory distress syndrome (HARDS) in the four corners region. Report to the Federal Centers for Disease Control and Prevention. July 1993. ‐ Publication No. 31. Sevilleta Long‐Term Ecological Research Program (LTER).

[ece33598-bib-0045] Pearson, O. P. (1966). The prey of carnivores during one cycle of mouse abundance. Journal of Animal Ecology, 35, 217–233. https://doi.org/10.2307/2698

[ece33598-bib-0046] Pearson, O. P. (1971). Additional measurements of the impact of carnivores on California voles (*Microtus californicus*). Journal of Mammalogy, 52, 41–49. https://doi.org/10.2307/1378430

[ece33598-bib-0047] Pearson, D. E. , & Callaway, R. M. (2006). Biological control agents elevate Hantavirus by subsidizing deer mouse populations. Ecology Letters, 9, 443–450. https://doi.org/10.1111/j.1461-0248.2006.00896.x 16623730 10.1111/j.1461-0248.2006.00896.x

[ece33598-bib-0048] Piudo, L. , Monteverde, M. , González Capria, S. , Padula, P. , & Carmanch, P. (2004). Distribution and abundance of sigmodontine rodents in relation to hantavirus in Neuquén, Argentina. Journal of Vector Ecology, 30, 119–125. 16007965

[ece33598-bib-0049] Prevedello, J. A. , Dickman, C. R. , Vieira, M. V. , & Vieira, E. M. (2013). Population responses of small mammals to food supply and predators: A global meta‐analysis. Journal of Animal Ecology, 82, 927–936. https://doi.org/10.1111/1365-2656.12072 23560951 10.1111/1365-2656.12072

[ece33598-bib-0050] R Core Team (2016). R: A language and environment for statistical computing. Vienna, Austria: R foundation for statistical computing Retrieved from http://www.R-project.org

[ece33598-bib-0051] Salisbury, F. (1992). Plant physiology, 4th ed Wadsworth, OH, USA: Belmont.

[ece33598-bib-0509] Schnurr, J. L. , Ostfeld, R. S. , & Canham, C. D. (2002). Direct and indirect effects of masting on rodent populations and tree seed survival. Oikos, 96, 402–410. https://doi.org/10.1034/j.1600-0706.2002.960302.x

[ece33598-bib-0510] Vieira, C. M. , Blamires, D. , Diniz‐Filho, J. A. F. , Bini, L. M. , & Rangel, T. F. L. V. B. (2008). Autoregressive modelling of species richness in the Brazilian Cerrado. Brazilian Journal of Biology, 68, 233–240. https://doi.org/10.1590/S1519-69842008000200003 10.1590/s1519-69842008000200003 18660950

[ece33598-bib-0052] Waltee, D. , Lonner, B. N. , Kuenzi, A. J. , & Douglass, R. J. (2009). Seasonal dispersal patterns of sylvan deer mice (*Peromyscus maniculatus*) within Montana rangelands. Journal of Wildlife Diseases, 45, 998–1007. https://doi.org/10.7589/0090-3558-45.4.998 19901376 10.7589/0090-3558-45.4.998 PMC2863021

[ece33598-bib-0053] Wolff, J. O. (1985). Comparative population ecology of *Peromyscus maniculatus*. –. Canadian Journal of Zoology, 63, 1548–1555. https://doi.org/10.1139/z85-230

[ece33598-bib-0054] Wolff, J. O. , & Sherman, P. W. (2008). Rodent societies: An ecological and evolutionary perspective. Chicago, IL, USA: University of Chicago Press.

[ece33598-bib-0055] Yates, T. , Mills, J. N. , Parmenter, C. A. , & Ksiazek, T. G. (2002). The ecology and evolutionary history of an emergent disease: Hantavirus pulmonary syndrome. BioScience, 52, 989–998. https://doi.org/10.1641/0006-3568(2002)052[0989:TEAEHO]2.0.CO;2

